# Identifying the challenges of policy content related to high-risk sexual behaviors, stimulant drugs, and alcohol consumption in adolescents

**DOI:** 10.1186/s12913-024-11256-w

**Published:** 2024-07-09

**Authors:** Saeid Mirzaei, Mohammad Hossein Mehrolhassani, Vahid Yazdi-Feyzabadi, Abbas Jahanara, Ali Akbar Haghdoost, Nadia Oroomiei

**Affiliations:** 1 Noncommunicable Diseases Research Center, Bam University of Medical Sciences, Sardaran Shahid Square- Shahid Rajaei Boulevard, Bam, Iran; 2 Medical Informatics Research Center, Institute for Futures Studies in Health, Kerman University of Medical Sciences, Kerman, Iran; 3 Health Services Management Research Center, Institute for Futures Studies in Health, Kerman University of Medical Sciences, Kerman, Iran; 4 Department of Pediatrics, School of Medicine, Pasteur Hospital, Bam University of Medical Sciences, Bam, Iran; 5 Health Modeling Research Center, Institute for Futures Studies in Health, Kerman University of Medical Sciences, Kerman, Iran

**Keywords:** High-risk sexual behaviors, Stimulant drugs, Alcohol consumption, Adolescents; Health policy

## Abstract

**Background:**

This study aims to identify policy content challenges related to high-risk sexual behaviors, stimulant drugs, and alcohol consumption in Iranian adolescents.

**Methods:**

This qualitative study analyzed high-level and national documents pertaining to adolescent health, high-risk sexual behaviors, stimulant, and alcohol consumption in adolescents. The documents, which were published by public organizations between January 1979 and February 2023 and publicly available, were complemented by interviews with policymakers and executives. The study involved reviewing 51 papers and conducting interviews with 49 policymakers and executives at the national, provincial, and local levels who were involved in addressing adolescent behaviors related to high-risk sexual behaviors, stimulant, and alcohol consumption. The data collected was analyzed using conventional content analysis.

**Results:**

The study’s results involved examining policy content and identifying challenges related to policy content. The analysis revealed that from the beginning of the Iranian revolution in 1979 until the late 1990s, the dominant approach in Iran was to deny the existence of high-risk behaviors among adolescents. However, in the early 2000s, the country began to adopt a new approach that acknowledged the social harms and ineffectiveness of previous strategies. As a result, a new policy framework was introduced to address high-risk behaviors among adolescents. The study’s interviews with policymakers and executives identified 12 challenges related to policy content, including parallel programs, lack of institutional mapping, lack of evidence-based policymaking, lack of integrated approach regarding training, late parent training, lack of consideration of all occurrence reasons in adolescents’ high-risk behaviors policymaking, and the existence of many abstinence policies regarding high-risk behaviors.

**Conclusions:**

The study’s findings suggest that high-risk behaviors among adolescents in Iran are primarily a health issue, rather than a social or ideological one. Unfortunately, ideological approaches, stigma, and policymaking based on anecdotes rather than evidence have had a significant impact on this area. To improve policymaking in this domain, it is crucial to address these challenges by tackling stigma, adopting an integrated and holistic approach, and implementing evidence-based policies that consider all relevant aspects, including adolescents’ subcultures and policy audiences. Such an approach can also be useful for other countries facing similar conditions.

**Supplementary Information:**

The online version contains supplementary material available at 10.1186/s12913-024-11256-w.

## Background

It is estimated that there are about 1.2 billion adolescents between the ages of 10 and 19 worldwide [1]. In the Middle East and North Africa (MENA) region, there are approximately 840,851 adolescents, according to the United Nations International Children’s Emergency Fund (UNICEF) [2]. Adolescence is a period of significant behavioral changes, during which new behaviors are formed that may not necessarily be healthy and can have a long-term impact on a person’s health. High-risk behaviors are among these behaviors [3, 4].

The studies conducted have indicated a high prevalence of high-risk behaviors among adolescents, including alcohol and substance use, especially stimulants [5–8]. These behaviors often occur together as they are co-occurring [9]. For instance, alcohol abuse in adolescents can reduce self-control, increase other high-risk behaviors, and lead to unprotected sexual relationships [3]. Globally, over one-fourth of all individuals between the ages of 15 and 19 (approximately 155 million people) consume alcohol. The prevalence of stimulant use is rapidly rising among adolescents and young adults. Substance use often begins during adolescence and early adulthood, and interventions during this critical period have the potential to impact the lifetime risk of developing stimulant use disorder and its associated negative effects [10]. It is important to note that high-risk behaviors among adolescents can have long-term negative impacts on their health and wellbeing, including reducing life expectancy. For example, complications related to pregnancy and childbirth are a leading cause of death for adolescent girls globally. In developing countries, approximately 12 million girls between the ages of 15 and 19 give birth each year [11]. The worldwide rate of adolescent delivery in 2020 was 43 births per 1000 girls in this age range, although this rate has decreased compared to 1990. To address this issue, countries have implemented various policies aimed at reducing high-risk behaviors among adolescents, including Iran [1].

Iran is located in the MENA region and is a country based on Islamic laws. Around 14% of its population is aged between 10 and 19 years old [12]. Islamic laws in Iran consider emotional relationships outside of marriage with the opposite sex as unacceptable and punishable by law [13]. Similarly, drinking alcohol is illegal and is also punishable by law, including whipping as a form of punishment. [14]. The consumption, selling, and buying of narcotic and stimulant drugs are also forbidden and punishable by law [14]. Despite these laws prohibiting high-risk behaviors such as extramarital sexual relationships [15], drugs [16], and alcohol [17], available data suggests a rising trend in these behaviors [18].

Considering that high-risk sexual behaviors, stimulant drugs, and alcohol consumption are crimes in Iran, and there is stigma attached to them, these behaviors occur clandestinely, and fear, concealment, and lack of transparency dominate the information in this area. On the other hand, adolescents, due to their more sensitive age, are at greater risk of experiencing these behaviors and potential harms. While several studies have investigated the prevalence and determinants of high-risk behaviors among Iranian adolescents, few studies have focused on identifying the challenges related to policy content.

Despite the policies implemented in this area, significant success in preventing and reducing harm from these behaviors among adolescents does not seem to be considerable. On the other hand, considering that policies are formulated in response to issues that have entered the agenda setting and if there are challenges in the adopted policies, the implementation and results of policy implementation will be affected. Therefore, the research question in the present study is: what are the challenges of policy content related to high-risk sexual behaviors, stimulant drugs, and alcohol consumption among adolescents in Iran? This will be addressed through reviewing policy documents and interviewing policymakers and implementers at national, provincial, and local levels. Identifying these challenges can provide valuable insights for future policy-making, not only in Iran but also in other countries facing similar conditions.

## Methods

The study involved a review of high-level and national documents related to adolescent health and high-risk behaviors, as well as interviews with policymakers and executives involved in addressing these issues at the national, provincial, and local levels in Iran. Document here means all the high-level and national documents related to adolescent health, high-risk sexual behaviors, stimulants, and alcohol consumption in adolescents written by public organizations published from January 1979 to February 2023 that are available to the public.

To identify the organizations involved in policy-making and implementing policies related to risky behaviors among adolescents, the ones mentioned in the “National Document for Reducing and Controlling Social Harms” in Iran were utilized [19]. This document identifies organizations related to high-risk behaviors and social harms. Based on this document, the research team examined websites and visited the organizations, requesting relevant documents. They also asked these organizations to introduce any others that have taken action in this area, resulting in a total of 71 documents. Of these, 20 documents were related to other high-risk behaviors of adolescents, which were excluded from the study. Next, three members of the team reviewed 51 relevant documents that addressed policies and executive programs related to these behaviors in adolescents. Of these documents, 21 were obtained through websites and the remaining 30 were obtained by visiting related organizations.

The organizations included Ministry of Education (MOE), Ministry of Welfare and Social Affairs, Ministry of Health, Welfare Organization, Supreme Council for Cultural Revolution, Presidential Organization, and Expediency Council’s website, Ministry of State, Judiciary, Parliament, Anti-narcotics Headquarters, Police Force, Islamic-Iranian Model Progress Center, Education Organization, Justice Organization, and General Department of Welfare. Related documents were selected based on Jupp quadruple considerations, i.e., reliability (being original and authentic), credibility (accuracy), representativeness (representative of all documents copies of the same classification), and meaning (what it says) [20]. A total of 51 documents were examined by the three members of research team, who carefully studied the records. Specifically, relevant sections of the documents that addressed policies and executive programs related to risky behaviors in adolescents were selected for analysis. The meaning of these sections was carefully analyzed, and the results of the examination were used to match and complete data gathered from interviews.

The study population includes all policy makers and executives involved in addressing these behaviors in adolescents, including those from the Ministry of Education, Ministry of Health and Medical Education, Welfare Organization, Anti-narcotics Headquarters, Anti-narcotics of the Expediency Council, Judiciary, and Ministry of Interior. The research sample included key policymakers and executives at three national, regional, and local levels of Kerman city (the center of Kerman Province as the biggest province of Iran). Participants were selected using a purposeful sampling method with a snowball strategy, which involved identifying initial participants based on predetermined criteria and then using their networks to identify additional participants. Participants were chosen based on predetermined criteria, including their knowledge of policies related to these behaviors, involvement in agenda-setting, planning, drafting, and executing procedures, and willingness to participate in interviews. Semi-structured interviews were conducted to collect data, and data saturation was achieved after conducting 49 interviews (Table 1).

**Table 1:** Number and position of interviewees

Level	Executive position	Number
National	Experts and officials of Social Harms Office of MOE	2
	Experts of Family and School Population Health Office of Ministry of Health and Medical Education	2
	Psychological, Social, and Addiction Health Office of Ministry of Health and Medical Education	3
	Experts and officials of Social Harms Prevention Office of Welfare Organization	2
	Officials of Cultural and Preventive Office of Anti-narcotics Headquarters	1
	Member of Independent Committee of Anti-narcotics of Expediency Council	1
Provincial (Kerman province, Iran)	Experts of Social Harms in General Provincial Department of Education	3
	Experts of Population, Family, and School Health Office of Kerman University of Medical Sciences, Iran	2
	Experts of Psychological, Social, and Addiction Health Office of Kerman University of Medical Sciences	2
	Experts and officials of Social Harms Prevention Office of Welfare Organization	2
	Social Deputy of the Police Force	2
	Experts of Social and Cultural Office of Provincial Government	2
	Experts of Social Deputy of Offence Prevention of Judiciary	2
	Experts of Cultural and Preventive Department of Coordination Council of Anti-narcotics Headquarters	1
	Chairman of Coordination Council of Anti-narcotics Headquarters of Kerman Province, Iran	1
Local (Kerman city, Iran)	School consultants	12
	School principals	4
	School teachers	3
	Experts of Social Harms Office of schools	2

Conventional content analysis was employed to scrutinize the data. In the document analysis section, documents identified according to Jupp’s criteria underwent repeated review by two members of the research team (N.O and S.M). Sections of the text pertaining to policies concerning high-risk sexual behaviors, stimulant drugs, alcohol consumption in adolescents, and policy challenges were identified and reviewed multiple times by the same two members. Based on the study’s objectives and an examination of adopted policies over time, alongside an assessment of whether documents addressed challenges to policy content, they were identified and coded.

Each interview was meticulously transcribed verbatim by two researchers (N.O and S.M) immediately after multiple listens. Simultaneous note-taking occurred during recording to document data throughout the research process. The selected texts and conducted interviews underwent careful scrutiny and analysis by the two research team members (N.O and S.M) to gain a comprehensive understanding of the content. Codes emerged from this analysis. The findings were discussed in a session attended by research members, and any discrepancies were addressed in a subsequent meeting attended by all members of the research team. The identified codes from document analysis and interviews were integrated, and final codes were determined. Document analysis served for data triangulation.

### Accuracy and robustness of data

To ensure credibility, the participants were given the opportunity to review and provide feedback on the findings. Additionally, the research team engaged in participatory thinking about the topics revealed at different stages of the study. Both interview and document analysis were used for data collection.

To ensure confirmability, the study documents were preserved throughout all phases of the research, and the findings were grounded in the data collected from the study participants. This was facilitated by several factors, including the researchers’ interest in the phenomenon under study, their prolonged engagement with the data, and their efforts to seek out others’ opinions. Additionally, the study was conducted by a team under the supervision and guidance of experts, which helped to verify its dependability and confirmability.

### Ethical considerations

It is important to note that this study was approved by the Bam University of Medical Sciences. Written consent forms were obtained from all participants prior to the interviews, which included permission for survey participation, voice recording, and transcription. It should be mentioned that this manuscript is part of a broader research project, which led to the publication of another article [21] based on a different part of the research.

## Results

The results of the present study included an examination of policy content and the identification of policy content challenges. Two periods, denial and acceptance, were observed in the policy content examination.

### Denial period

From the beginning of the Iranian revolution in 1979 until the late 1990s, the dominant approach was the denial of high-risk sexual behaviors, stimulant drugs, and alcohol consumption. Despite reports of substance use disorder among students, the Comprehensive Prevention of Addiction Document in the mid-1990s emphasized the reluctance and resistance within educational organizations to implement substance use prevention programs for adolescents. For example, the documents emphasize the reinforcement of adolescents’ resilience against cultural invasion and social vices [22]. Additionally, they underscore the importance of creating a conducive environment for the development of ethical virtues, with the government taking on the responsibility of combating manifestations of corruption and decadence [23]. It is imperative to mobilize all resources within executive bodies to effectively eradicate social vices [24]. This conflict continued until 1999, when studies about substance use prevalence in schools were conducted, indicating that, unlike the MOE’s management assumption, substance use prevalence was increasing. Despite the establishment of the Drug Abuse Prevention Office in the MOE in 1999, the MOE insisted that the introduction of topics related to substance use in education did not indicate an expansion of substance use. Still, it was a precautionary measure taken to prevent potential danger. However, the statistics showed substance use expansion among students. The contributions of the Drug Abuse Education and Prevention Department of the MOE were based on training, little research, and a life skills training program; however, no change was made.

As a result, policy content in those years considered avoiding the effects of Western vulgar culture as the solution for eradicating these behaviors. Therefore, resistance against the majority of abnormal behavioral patterns and legal and spiritual obligations to maintain purity and avoid ethical corruption were the solutions.

### Acceptance period

In the early 2000s, Iran entered a new era. The acknowledgment of risky behaviors, such as substance use disorders, and the recognition of the ineffectiveness of previous strategies became apparent. Although the denial approach still persisted, various primary and tertiary prevention policies were implemented during this period. For example, the documents mention the implementation of intervention plans for social harms, particularly targeting students at risk and in high-risk situations [25]. Additionally, there is a reference to referring adolescents to mental health centers for substance cessation with a harm reduction approach [26]. Regarding high-risk sexual behaviors, the documents underscore ethical commitments to uphold modesty, chastity, and avoid moral corruption. They also emphasize the promotion of modesty and veiling to prevent relationships outside the framework of marriage [27–29].

### Primary prevention

In the realm of adolescent programs and initiatives, the primary focus is on implementing direct prevention strategies. These strategies include education for adolescents, fear appeals, promotional and cultural activities, parental education on risky behaviors, and school staff education on risky behaviors.

### Education for adolescents

A paramount emphasis in prevention efforts lies in educating adolescents. Key stakeholders in this field include educational organizations, the judiciary, the Ministry of Health, the police force, and the Welfare Organization. The educational content for adolescents encompasses life skills education, self-care education, and socio-psychological empowerment.

### Fear appeals

After education, the primary emphasis turns to conveying fear appeals regarding substance abuse, alcohol consumption, and risky behaviors. A substantial portion of these messages is delivered with a religious perspective, primarily by educational organizations, the judiciary, and the police force.

### Promotional and cultural activities

Another crucial aspect of primary prevention involves promotional and cultural activities. These activities include exhibitions, festivals, and the production of cultural products aimed at raising public awareness and promoting knowledge and understanding of risky behaviors. They also encourage participation to promote healthy lifestyles and prevent risky behaviors and social harms. Educational organizations, the Anti-Drug Headquarters, and the Welfare Organization are responsible for these activities.

### Parental education on risky behaviors

Parental education activities for primary prevention include training in parenting skills, family life skills, recognition of risky behaviors and risk factors in adolescents, understanding growth changes and adolescence, recognizing adolescents at risk, and understanding protective and risky factors within families. Educational organizations are the primary drivers of initiatives in this area.

### School staff education on risky behaviors

Another critical preventive measure involves training school staff in the realm of social harms and risky behaviors. This training includes recognizing risky behaviors and risk factors, identifying adolescents at risk, understanding adolescence, recognizing protective and risk factors for adolescents in schools, and teaching effective communication strategies with adolescents. The Ministry of Education is responsible for implementing this program.

### Tertiary prevention

A limited number of programs adopt the tertiary prevention approach, which includes the provision of psychiatric services for quitting substance abuse and alcohol, as well as counseling and referral for specialized services.

### Provision of psychiatric services for quitting substance abuse and alcohol

In the domain of tertiary prevention, adolescents identified with substance abuse or alcohol misuse are directed to psychiatric centers for services, including outpatient, daily, or inpatient care, to undergo cold turkey cessation. This referral process is coordinated with the Anti-Drug Headquarters.

### Counseling and referral for specialized services

Another policy in the realm of tertiary prevention involves counseling and referral for specialized diagnostic and therapeutic services for HIV/AIDS and other infectious diseases. This program is primarily available at urban health facilities. Adolescents who voluntarily visit these facilities and exhibit risky sexual behavior during initial assessment are referred for specialized services voluntarily. The Ministry of Health oversees these initiatives.

### Challenges of policy content

In the domain of policy content, twelve content challenges have been identified and are addressed in Table 2.

**Table 2:** Challenges of policy content

Challenges of policy content	Existence of parallel programs
	Lack of institutional mapping
	Lack of evidence-based policymaking
	Lack of an integrated approach for training
	Late parent training
	Lack of consideration of all occurrence reasons for adolescents’ risky behaviors in policymaking
	Lack of consideration of adolescents’ family conditions in policy content
	Lack of specific training content in judiciary training
	Lack of lessons related to high-risk behaviors in the curriculum of Farhangian University
	Criminalization of drug and alcohol consumption and secrecy
	Emphasis on abstinence policies regarding high-risk sexual behaviors
	Insufficient attention to the rehabilitation domain

### Existence of parallel programs

The presence of numerous programs with life skill educational content in the Ministry of Education, as well as various parallel educational programs offered by different organizations for the adolescent target group, pose a significant challenge. This situation results in a reduction of training quality and resource waste.

As one participant (I10) expressed, “*Here in the Education Organization, we have several programs that essentially say the same thing. However, every time someone comes up with a new program that is similar to the previous ones, they suggest executing it. The schools’ capacity for execution is limited, and it is impossible to carry out all these programs with good quality*.”

Another participant (I12) said, “*There are too many programs, and new ones are added every day. Our schools lack the capacity to handle them all. The responsibilities for these programs fall on the principals and other educational personnel, and the costs associated with them are substantial. If these programs were designed with the schools’ capacity in mind, we could have 2 or 3 programs instead of many, and we could execute them well.”*

“*’I have repeatedly pointed out in headquarters meetings that having 40 programs for one organization is not a practical or controllable approach* “(I14).

### Lack of institutional mapping

One of the key challenges in policy content is the lack of institutional mapping, which refers to the absence of a roadmap that illustrates the leading roles, their interactions, current resources, and proceedings to determine potential obstacles. This issue often causes organizations to change their selected policies and proceedings to what they can do rather than what they must do.

As one participant (I9) pointed out, *“We need to establish institutional mapping first, and then we can identify what we have done, where we would like to go, and what each organization must do. However, this does not happen in practice. When we create policies, officials from various organizations attending the meetings often ask who can take action to prevent risky behaviors. One organization may claim that it can handle the job delegated to them, but this should not be the case. As a result, we see that organizations such as Education, Welfare, Police, or Judiciary are providing training because, in their opinion, it is the easiest thing to do.”*

### Lack of evidence-based policymaking

An analysis of documents and interviews revealed that the lack of evidence-based policymaking is a significant challenge in policy content. A significant proportion of training programs addressing high-risk behaviors, such as drug and alcohol consumption, rely on fear appeals to deter adolescents. However, evidence suggests that the effectiveness of these messages in preventing such behaviors depends on various factors that should be considered. On the other hand, there exist subcultures among adolescents, and taking these into account when designing policy content can improve the acceptance of policies and align them better with the reality of the adolescent world.

“*As one participant (I22) noted, ‘The Education Organization hired university professors to teach students about HIV, for example, but this is not an effective approach as it lacks references. Their way of teaching is based on fear appeal, but the effectiveness of these messages depends on various factors. Such programs are not allowed in primary and secondary schools, and they only start in high schools, where students are less receptive to such messages and often just laugh at them. Holding workshops at schools to earn money is a matter of pride for schools, but, in reality, such workshops often yield no results.*”

### Lack of an integrated approach for training

The prevention domain puts significant emphasis on training adolescents; however, there is no single approach to training, as it is affected by general country policies. Public policies, in turn, are influenced by ideology, secrecy, and a tendency to consider any discussion about high-risk sexual behaviors as taboo. As a result, the focus of training is often misguided and fails to address the issue adequately. Some policymakers believe that acknowledging the existence of these behaviors and providing appropriate training and harm reduction strategies is necessary. However, others believe that denying the problem, avoiding discussion, and not addressing it directly is the solution.

One interviewee highlighted the lack of actual educational content in current training programs, stating, “*Many of our training is not training, i.e., no actual educational content. The training is based on ideology, secrecy, and considering the behaviors taboo. They are based on creating a sense of guilt; therefore, they are not feasible*” (I21).

On the other hand, some interviewees believe that discussing these issues openly and providing training can have negative consequences. One interviewee stated, “*I believe this training is inappropriate, and we must not talk about these behaviors because these are taboos and should be regarded as taboo. There were no such behaviors when we were students because we did not dare to be impolite at school. We respected the teachers and did not discuss drugs and alcohol in class. We cannot control these students; these problems all began when psychology entered the education domain; in the past, the mothers used a stick to punish the children, and all of them were polite and did not dare to do such things. When we imitate the westerns, their problems will infect us*” (I25).

### Late parent training

One of the challenges in policy content is late parent training, where parents are trained on how to deal with high-risk behaviors of adolescents and parenting styles after their children have already reached maturity. Ideally, such training should be provided long before the child becomes mature or even before the parents decide to have a child so that they can be effective. However, teaching parenting styles to parents of adolescents may not be adequate.

“*The parents have a 15-year-old adolescent whom they have been educating in their own way. When the child turns 15, the education organization tells the parents to empower themselves in dealing with their adolescent. But can the parents really change themselves, or can we change the adolescent’s character? It seems like it’s just for show and won’t make much of a difference.*” (I15).

In other words, the training may come too late for parents to change their parenting style or for the adolescents to change their behavior. This underscores the importance of early intervention in parenting and providing resources to parents at an earlier stage.

### Lack of consideration of all occurrence reasons of adolescents’ risky behaviors in policy-making

There are multiple reasons why high-risk behaviors occur among adolescents, but the policies aimed at preventing such behaviors often fail to consider all of these reasons.

“*Scientific evidence shows that many factors contribute to the occurrence of high-risk behaviors, including family environment and early childhood experiences. However, policymakers often overlook these factors and focus on simple reasons. For instance, the mother-child relationship in the first 1000 days of life plays a significant role in shaping the child’s behavior. If the child is frequently separated from the mother during this period, insecure attachment may develop, which can probably lead to high-risk behaviors later in life. Unfortunately, policymakers often disregard evidence, and, for instance, they neglect to establish a structure that enables working mothers to stay with their children until they reach three years old—the critical first 1000 days of life. Instead, they train parents when their adolescents are in high school, which is too late. Such training is often ineffective and only benefits the trainers who earn money from it*” (I5).

### Lack of consideration of adolescents’ family conditions in policy content

The interviewees believed that the current policies are useless and could not cover the adolescents and their families because some adolescents with high-risk behaviors have high-risk parents. For instance, an interviewee stated:

*“For example, when adolescents who abuse drugs or alcohol are sent to a psychological center for withdrawal, they may quit with a lot of effort, but they may return to their family with addicted parents and revert to their old ways. I had a student who quit heroin five times, but their parents were addicted to heroin and methamphetamine, and they eventually lost their energy to keep fighting. Why do policymakers ignore the parents of such adolescents, and why isn’t anything done to ensure their custody?”* (I2).

### Lack of specific training content in judiciary trainings

The judiciary has a program that trains life skills for juvenile delinquents. The interviewees in the judiciary believe that although the educational topics are determined by the Cultural and Crime Prevention Deputy of the Judiciary at the national level, the content of each chapter is not predetermined, and the psychologist who is in charge of the training determines the educational content based on their expertise.

“For *instance, the training is supposed to teach self-esteem, but the content is not clearly defined. The psychologist decides what, how, and how many sessions to conduct. If our psychologist lacks experience, the quality of training will be subpar and not yield the expected results”* (I19).

### Lack of lessons related to high-risk behaviors in curriculum of Farhangian University

Farhangian University is a university that trains teachers, and after graduation, they start working in Iran’s schools. Although the teachers have an essential role in preventing the occurrence of these behaviors, and the students of this university are the future teachers, attention is not paid to the lessons related to high-risk behaviors in the curriculum.

“*The students of Farhangian University, who have education organization scholarships, are committed to teaching in our schools. They have too many religious lessons but no lessons related to high-risk behaviors. They hope to change the teachers’ beliefs with several in-service training sessions and draw attention to high-risk behaviors. However, this seems impossible as the training should have happened much earlier. They must understand what high-risk behavior is and recognize the signs in students. Additionally, they should be aware that a student may be in danger, but no training has been provided. If problems were supposed to be solved by teaching religious topics, that would happen. Therefore, education organizations must change their approach, but unfortunately, they do not.”* (I2).

### Criminalization of drug and alcohol consumption and secrecy

Another challenge is the criminalization of drug and alcohol consumption, which is exacerbated by certain training programs, such as the ‘judge at school program’ where a judge from the Judiciary explains high-risk behaviors from a legal viewpoint to students. This increases secrecy about these behaviors among adolescents, which can lead to the late identification of the issue and cause more harm to them.

“*It’s usually difficult for the students to like me as their teacher at school. So, sometimes we bring in a judge to explain to them the legal consequences of carrying drugs and getting arrested by the police. However, this has led to students becoming more secretive, which only makes the problem worse. We are not trying to solve the problem; we are just trying to hide it from view. In fact, we haven’t even evaluated the effectiveness of the ‘judge at school program since 2013*!”

### Emphasis on abstinence policies regarding high-risk sexual behaviors

Although policies regarding high-risk behaviors have entered the acceptance stage, there is still a denial approach in the policymaking domain, as evidenced by document examination and interviewees’ opinions. The central policies in sexual behaviors focus on abstinence and chastity considerations. However, evidence shows that there are three strategies for dealing with high-risk sexual behaviors: abstinence, having no more than one sexual partner, and using a condom. Policymakers have paid almost no attention to those who do not accept the abstinence policy and have not provided comprehensive training for the other two strategies. This can have a significant impact on HIV transmission in the future in Iran, given the changing patterns of HIV transmission in sexual communication.

“*We tend to deny the existence of sexual topics. The reality is that there are many cases of sexual relationships before marriage without any training about sexually transmitted diseases and dangerous sexual behavior. However, our policymakers are unwilling to acknowledge this issue; they refuse to talk about it…”* (I36).

## Insufficient attention to rehabilitation domain

The third level of prevention, rehabilitation, which involves reducing the harms resulting from these behaviors in adolescents, is often overlooked, which can impact the effectiveness of prevention policies.

“*’The next point is that no policy regarding rehabilitation was considered. We have done nothing when we do not help rehabilitate the behaviors accompanying harm and stigma in our society and the policies that do not complete their prevention cycle. The drug-abusing adolescent quits and comes back again to society. The family is also engaged in drug abuse, and the adolescent returns to the same family. After a while, we must help him again to withdraw” (I17).*

## Discussion

The policy content concerning high-risk behaviors, drugs, and alcohol consumption among Iranian adolescents has undergone two phases of denial and acceptance. This is because policymaking in this area has been influenced by Iran’s policy conditions regarding social harms and high-risk behaviors [21]. Similarly, Khodayari-Zarnaq & et al. referred to policymaking conditions based on denial and, to some extent, acceptance regarding HIV/AIDS in Iran in their study [30].

During the acceptance phase, policies employing primary and tertiary prevention approaches were implemented. In the field of primary prevention, actions were taken, although there were discussions regarding implementation methods. For example, family-related factors have proven to be efficient and effective protective elements in this realm [31, 32]. Van Raizin et al. also identified family-based interventions as the most effective means of preventing high-risk behaviors in adolescents [33]. However, interviewees reported that family education, awareness, and empowerment strategies were not at desirable levels.

In the realm of tertiary prevention, efforts have been made to address these behaviors. For example, in the area of stimulant drug and alcohol consumption, effective therapy and recovery for adolescents require a comprehensive approach, as indicated by several studies. This approach involves evaluating and intervening at different stages of relapse, such as precontemplation, contemplation, decision-making, withdrawal, and assessing substance-related disorders [34]. Detoxification tailored to individual needs, family therapy, group therapy, and additional support services like education and vocational training are crucial. However, abstaining from alcohol and stimulants and preventing relapse pose significant challenges [35]. A study by Benton demonstrated that cognitive-behavioral therapy has shown promise in reducing relapse likelihood, although adolescents’ motivations for substance use differ from those of adults. Successful therapy outcomes hinge on understanding the context of substance abuse in adolescents [36].

In addressing high-risk sexual behaviors, strategies such as practicing abstinence, having only one sexual partner, getting tested for HIV, and using condoms are advocated [37]. However, in Iran, abstinence is the prevailing strategy [38]. Health centers offer counseling and referrals for adolescents engaging in high-risk behavior, but sexual activity outside of marriage is stigmatized, potentially deterring adolescents from seeking help [39]. Furthermore, there is limited support for adolescents who do not access these centers, increasing the risk of sexually transmitted diseases transmission, including HIV.

The content of policies related to adolescent behavior faces several challenges, including a lack of evidence-based policymaking, delayed parent training, insufficient consideration of all the reasons behind adolescents’ risky behaviors in policymaking, and a lack of respect for the family conditions of adolescents. Research indicates that parental involvement and the first 1000 days of a child’s life are critical factors influencing behavior [40–45]. Parenting style also plays a significant role in behavior development [31, 35, 46, 47]. Scientific evidence highlights the profound impact of early childhood experiences, with family dynamics shaping an individual’s character [48]. Therefore, interventions and training for parents before or during early stages of a child’s life are suggested to be more effective in addressing behavioral issues.

The lack of evidence-based policymaking and neglecting adolescent subcultures present considerable challenges in policymaking. Incorporating knowledge of these subcultures can enhance policy acceptance and lead to more relevant and effective policies. Recognizing adolescent subcultures can result in policies that better reflect their experiences and perspectives. Numerous studies have highlighted the importance of considering adolescent subcultures when crafting policy solutions to address their issues [49–52].

Evidence-based policymaking relies on scientific evidence rather than anecdotes [53]. An alternative approach, evidence-making intervention, recognizes contextual differences and emphasizes that interventions can generate evidence for policymaking [54–56]. However, the effectiveness of interventions should be prioritized, regardless of the approach taken. Assessing interventions based on scientific evidence, not anecdotes, is crucial, highlighting the need for further investigation in this field [53–56].

Another challenge is the absence of institutional mapping and an integrated approach in training and parallel programs. A study by Zhou et al. indicated that this fragmented approach, particularly prevalent in low- and middle-income countries, results in disjointed actions and conflicting programs. Addressing these challenges requires a more cohesive and integrated approach to policymaking [57].

Another challenge is the lack of lessons related to high-risk behaviors in the curriculum of Farhangian University, despite the significant role of teachers in preventing the occurrence and timely identification of high-risk behaviors in students [58]. The study by Mzingwane et al. highlighted the essential role of teachers in prevention training and reducing the harm caused by high-risk behaviors in adolescents in Zimbabwe [59].

## Conclusions

This study highlights the policy landscape surrounding high-risk behaviors among Iranian adolescents, emphasizing the need for evidence-based, comprehensive policies. Neglecting the social nature of these behaviors in policymaking can lead to ineffective strategies. Despite growing recognition, some policymakers still deny their prevalence, impacting policy effectiveness. Moving forward, adopting evidence-based policies and addressing family conditions are imperative. This study fills a critical gap in research, providing insights into policymaking challenges.

Furthermore, the findings have broader implications beyond Iran, informing efforts to promote adolescent health globally. The study underscores the importance of incorporating evidence into policymaking. Religious, traditional, and cultural approaches alone are insufficient for prevention, treatment, and rehabilitation, and can lead to stigma and concealment of these behaviors.

Health policymakers must adopt scientific, evidence-based approaches that consider all contributing factors, including the target age group. Recognizing and accepting these behaviors as potential issues in human society, rather than viewing them as sins or moral vices, along with monitoring their prevalence, can influence policymakers’ strategies. Effective prevention at various levels requires institutional mapping with a clear division of tasks to avoid superficial and parallel programs lacking scientific support. Poorly designed policies and actions can significantly damage the perceived importance of the issue, the interventions made, and the trust of the policy’s audience.

Focusing on family structure, early education on high-risk behaviors, training teachers during their student years and through in-service programs based on scientific evidence with precise educational content, decriminalization, harm reduction, and comprehensive prevention strategies can help mitigate challenges in this domain.

### Electronic supplementary material

Below is the link to the electronic supplementary material.

Supplementary Material 1

Supplementary Material 2

## Data Availability

The datasets used / analyzed during the current study are available from the corresponding author on reasonable request.

## Abbreviations

MOE: Ministry of Education MOE
