# Supplementary material for: Identifying the challenges of policy content related to high-risk sexual behaviors, stimulant drugs, and alcohol consumption in adolescents
Source: BMC Health Serv Res. 2024 Jul 9;24:788. doi: 10.1186/s12913-024-11256-w (PMC11234520; doi:10.1186/s12913-024-11256-w)
Supplement: Supplementary file 1 — Supplementary Material 1 [file 12913_2024_11256_MOESM1_ESM.docx]

**Plain English summary**

Iran is an ideological country based on Islamic laws, in which every emotional relationship outside marriage, drugs, and alcohol consumption is considered an offense. However, studies show the increasing trend of these behaviors in the Iranian context, especially among adolescents. Therefore, this qualitative study aims to identify policy content challenges related to high-risk sexual behaviors, stimulant drugs, and alcohol consumption in Iranian adolescents. The analysis revealed that from the beginning of the Iranian revolution in 1979 until the late 1990s, the dominant approach in Iran was to deny the existence of high-risk behaviors among adolescents. However, in the early 2000s, the country began to adopt a new approach that acknowledged the social harms and ineffectiveness of previous strategies. The challenges related to policy content, including parallel programs, lack of institutional mapping, lack of evidence-based policymaking, lack of integrated approach regarding training, late parent training, lack of consideration of all occurrence reasons in adolescents' high-risk behaviors policymaking, and the existence of many abstinence policies regarding high-risk behaviors were identified. These behaviors are primarily a health issue, rather than a social or ideological one. Unfortunately, ideological approaches, stigma, and policymaking based on anecdotes rather than evidence have significantly impacted this area. To improve policymaking in this domain, it is crucial to address these challenges by tackling stigma, adopting an integrated and holistic approach, and implementing evidence-based policies that consider all relevant aspects, including adolescents' subcultures and policy audiences. Such an approach can also be useful for other countries facing similar conditions.
