# Supplementary material for: Identifying the challenges of policy content related to high-risk sexual behaviors, stimulant drugs, and alcohol consumption in adolescents
Source: BMC Health Serv Res. 2024 Jul 9;24:788. doi: 10.1186/s12913-024-11256-w (PMC11234520; doi:10.1186/s12913-024-11256-w)
Supplement: Supplementary file 2 — Supplementary Material 2 [file 12913_2024_11256_MOESM2_ESM.docx]

**Data Collection and Relationship to Previous Work**

**Data Collection**

The data for this study were collected through structured interviews conducted with policy makers and executives in adolescent health. The interview questions were specifically developed for this research to explore various aspects of policies addressing risky sexual behaviors, stimulant use, and alcohol abuse among adolescents.

**The interview questions were as follows:**

1. What topics have led to risky sexual behavior, stimulants, and alcohol abuse among adolescents to be considered as a problem in policy-making?
2. Which organizations or groups have helped bring this issue to the agenda?
3. What policies and solutions have been proposed to solve the problem?
4. Which of these policies have received attention and acceptance, and which ones have not? What was the reason for this lack of attention and acceptance?
5. Which organizations, groups, or even individuals have played a role in presenting and accepting existing policies as a political solution?
6. What events have led to these actions being presented and accepted as policy solutions, and policy documents being developed?
7. What policies are currently in place to address this issue among adolescents?
8. What is the content of these policies?
9. In your opinion, what are the challenges of the content of these policies?

**Relationship to Previous Work**

This manuscript focuses on analyzing the data obtained from questions 7 to 9, which investigate the current policies addressing risky behaviors among adolescents, their content, and the challenges associated with them.

Previously, an article titled "Agenda-setting in policies related to high-risk sexual behaviours, stimulants, and alcohol abuse in Iranian adolescents" was published based on the analysis of data from questions 1 to 6 (1). This prior work examined the factors that led to the inclusion of these issues on the policy agenda and the initial responses from various stakeholders.

Both articles stem from the same dataset but address different research questions and aspects of the issue. The current manuscript builds upon the findings of the previous study by delving into the specifics of existing policies and their implementation challenges. The previous publication has been referenced in this manuscript to provide context and acknowledge the foundation it laid for the current analysis.

**Ethical Considerations**

The division of the research into two publications was done to provide a detailed and focused analysis of distinct aspects of the data. The first article addressed the agenda-setting process and stakeholder involvement, while the current manuscript focuses on the content and challenges of existing policies. Each manuscript provides unique insights and contributes to a comprehensive understanding of the issue. The approach taken ensures that the findings are presented in a clear and structured manner, allowing for a deeper exploration of each aspect.

By transparently outlining the data collection process and the relationship between the two articles, we aim to uphold the integrity of the research and provide a clear framework for readers to understand the contributions of each study.

**References:**

1. Mirzaei S, Mehrolhassani MH, Yazdi-Feyzabadi V, Haghdoost A, Oroomiei N. Agenda-setting in policies related to high-risk sexual behaviours, stimulants, and alcohol abuse in Iranian adolescents. Heal Res policy Syst. 2023;21(1):104.
